# Prevalence of malnutrition in Saudi children: a community-based study

**DOI:** 10.4103/0256-4947.67076

**Published:** 2010

**Authors:** Mohammad I. El Mouzan, Peter J. Foster, Abdullah S. Al Herbish, Abdullah A. Al Salloum, Ahmad A. Al Omar, Mansour M. Qurachi

**Affiliations:** a From the Department of Pediatrics, King Saud University, Riyadh, Saudi Arabia; b From the School of Mathematics, Manchester University, Manchester, UK; c From the The Children’s Hospital, Riyadh Medical Complex, Riyadh, Saudi Arabia; d From the Department of Pediatrics, Al Yamama Hospital, Riyadh, Saudi Arabia

## Abstract

**BACKGROUND AND OBJECTIVE:**

There is no published information on the prevalence of malnutrition in Saudi Arabia. The objective of this study was to establish the prevalence data.

**METHODS:**

The prevalence of nutritional indicators in the form of underweight, stunting, and wasting in a national sample of children younger than 5 years of age was calculated using the new WHO standards as reference. Calculations were performed using the corresponding WHO software. The prevalence of moderate and severe underweight, wasting and stunting, was defined as the proportion of children whose weight for age, weight for height, and height for age were below –2 and –3 standard deviation scores, respectively.

**RESULTS:**

The number of children younger than 5 years of age was 15 516 and 50.5% were boys. The prevalence of moderate and severe underweight was 6.9% and 1.3%, respectively. The prevalence of moderate and severe wasting was 9.8% and 2.9%, respectively. Finally, the prevalence of moderate and severe stunting was 10.9% and 2.8%, respectively. The prevalence was lower in girls for all indicators. Comparison of the prevalence of nutritional indicators in selected countries demonstrates large disparity with an intermediate position for Saudi Arabia.

**CONCLUSION:**

This report establishes the national prevalence of malnutrition among Saudi children. Compared to data from other countries, these prevalence rates are still higher than other countries with less economic resources, indicating that more efforts are needed to improve the nutritional status of children.

The nutritional status of children is one of the well-known indicators of economic development. This has been stressed as one of the eighth Millennium Development Goals adopted by the United Nations in 2000.^1^ Growth indices in the form of weight for age, weight for height, and height for age are important tools for the assessment of nutritional status of children.^2^ The prevalence of nutritional indicators in the form of underweight, stunting, and wasting in children under 5 years of age is one of the ways of assessment of nutritional status of the population. Surveillance of these indicators is commonly practiced regularly in many countries as a measure of public health status. The available data indicate that the prevalence of nutritional indicators is lowest in Western countries such as the United States and highest in much less developed countries such as Bangladesh and Yemen, with an increasing number of countries called “transitional countries” such as Chile and Brazil in which gradual economic development resulting in steady improvement of health has been reflected in marked reduction of the prevalence of nutritional indicators.^3^

In Saudi Arabia, despite previous publications of anthropometry in children,^4^ data reflecting the national prevalence of malnutrition in Saudi children are not available in the World literature. The objective of this report is therefore to establish the national prevalence of nutritional indicators (underweight, wasting and stunting) in Saudi children.

## METHODS

The prevalence of underweight, stunting, and wasting in Saudi children from birth to 5 years was calculated using data sets from the 2005 growth charts for Saudi children and adolescent survey. The design and methodology of that study have been reported in detail elsewhere.^5^ In summary, the sample size was calculated according to standard guidelines and criteria.^6^ The study sample was selected by the multistage probability sampling procedure from a stratified listing based on the population census in Saudi Arabia. Accordingly, the sample is representative of all the socioeconomic strata and consists of a majority of children with mixed breast milk and artificial formula feeding. Data collection was made by house-to-house visits where a survey questionnaire, clinical examination, and body measurements were completed by primary care physicians and nurses. Data were analyzed using the new Lambda, Mu, Sigma (LMS) Methodology.^7^–^9^ The prevalence of underweight, stunting, and wasting in infant and children under 5 years of age was calculated using the published World Health Organization (WHO) software.^10^ The prevalence of moderate and severe underweight was defined as the proportion of children whose weight for age was below –2 and –3 standard deviation scores (SDS), respectively. Similarly, the prevalence of moderate and severe wasting and stunting was defined as the proportion of children with weight for height (wasting) or height for age (stunting) were below –2 and –3 SDS, respectively. All calculations were performed using the new WHO child growth standards.^11^ The term height will be used throughout the manuscript to include length/height for simplification.

## RESULTS

The number of children younger than 5 years of age was 15 516 and 50.5% were boys. The prevalence of moderate (6.9%) and severe underweight (1.3%) is presented inTable 1 and indicates a progressive increase in the prevalence of underweight with advancing age, with girls consistently having lower prevalence than boys.Table 2 depicts a prevalence of wasting of 9.8% and 2.9% for moderate and severe wasting, respectively, with an inconsistent pattern according to age, but with a consistently lower prevalence of wasting in girls. Finally,Table 3 shows a prevalence of stunting of 10.9% and 2.8% for moderate and severe degrees, respectively. Although the overall prevalence was lower in girls, this pattern was not consistent according to age.Table 4 summarizes a comparison of the prevalence of nutritional indicators among selected countries from most continents displaying the disparity between children in other countries and Saudi children.

**Table 1:** Prevalence of underweight by age and gender.

Age (years)	Total number (%) <–2 SD			Total number (%) <–3 SD		
	Boys	Girls	Combined	Boys	Girls	Combined
<1	4343 (5.1)	4141 (2.3)	8484 (3.7)	4343 (1.1)	4141 (0.5)	8484 (0.8)
1 to < 2	1283 (5.5)	1228 (2.9)	2511(4.2)	1283 (0.8)	1228 (0.4)	2511 (0.6)
2 to < 3	703 (7.8)	727 (7.2)	1430 (7.5)	703 (1)	727 (1.5)	1430 (1.3)
3 to < 4	716 (7.8)	787 (8.8)	1503 (8.3)	716 (2)	787 (1.5)	1503 (1.8)
4 to < 5	845 (10.3)	828 (11.1)	1677 (10.7)	845 (2.1)	828 (1.6)	1673 (1.9)

**Overall**	**7890 (7.3)**	**7711 (6.5)**	**15601 (6.9)**	**7890 (1.4)**	**7711 (1.1)**	**15 601 (1.3)**

**Table 2:** Prevalence of wasting by age and gender

Age (years)	Total number (%) <–2 SD			Total number (%) <–3 SD		
	Boys	Girls	Combined	Boys	Girls	Combined
<1	4290 (12.3)	4109 (10.8)	8399 (11.6)	4290 (5.6)	4109 (4.4)	8399 (5)
1 to < 2	1282 (6.9)	1227 (4.5)	2509 (5.7)	1282 (1.9)	1227 (1.6)	2509 (1.8)
2 to < 3	698 (12.3)	727 (8.4)	1425 (10.4)	698 (3.2)	727 (2.8)	1425 (3)
3 to < 4	714 (10.5)	785 (9)	1499 (9.8)	714 (2.5)	785 (1.9)	1499 (2.2)
4 to < 5	893 (12.6)	826 (10.8)	1719 (11.7)	893 (3.5)	826 (1.9)	1719 (2.7)

**Overall**	**7877 (10.9)**	**7674 (8.7)**	**15551 (9.8)**	**7877 (3.3)**	**7674 (2.5)**	**15 551 (2.9)**

**Table 3:** Prevalence of stunting by age and gender.

Age (years)	Total number (%) <–2 SD			Total number (%) <–3 SD		
	Boys	Girls	Combined	Boys	Girls	Combined
<1	4336 (10.5)	4123 (5.2)	8459 (7.9)	4336 (3.3)	4123 (1.5)	8459 (2.4)
1 to < 2	1278 (12.5)	1225 (11.1)	2503 (11.8)	1278 (3.8)	1225 (3.3)	2503 (3.6)
2 to < 3	703 (14.1)	726 (10.1)	1429 (12.1)	703 (3.4)	726 (3.4)	1429 (3.4)
3 to < 4	716 (11.7)	787 (12.8)	1503 (12.3)	716 (3.4)	787 (2.5)	1503 (3.0)
4 to < 5	845 (10.1)	829 (10.3)	1674 (10.2)	845 (1.8)	829 (1.9)	1674 (1.9)

**Overall**	**7878 (11.8)**	**7690 (9.9)**	**15568 (10.9)**	**7878 (3.1)**	**7690 (2.5)**	**15 568 (2.8)**

**Table 4:** Comparative national prevalence of nutritional indicators in selected countries.

Country	Study years	Sample size	Underweight (%)		Wasting (%)		Stunting (%)	
			<–2 SD	<–3 SD	<–2 SD	<–3 SD	<–2 SD	<–3 SD
Bangladesh^19^	2006	24 302	39.8	10.3	12.4	1.6	47	15.5
Yemen^20^	2003	12 364	43.1	18.5	15.2	6.3	57.7	35.8
Brazil^21^	2006-07	4415	2.2	0.3	4.0	1.6	7.1	1.5
USA^17^	1999-04	3920	1.3	0.2	0.6	0.1	3.9	0.5
Egypt^22^	2005	12 830	5.4	1.7	5.3	2.5	23.8	10.3
Nigeria^23^	2003	5407	27.9	11.9	11.2	4.8	43	23.7
Indonesia^24^	2007	77 808	19.6	5.1	14.8	6.8	40.1	21
Oman^25^	1999	14 076	11.3	1.6	7.3	1.1	12.9	2.4
Present study	2004-05	15 601	6.9	1.3	9.8	2.9	10.9	2.8

## DISCUSSION

Previous estimates of the prevalence of malnutrition in the world used older references such as the National Center for Health Statistics (NCHS)/World Health Organization (WHO) based on the population from the United States and adopted by the WHO,^12^,^13^ and the 2000 Center for Disease Control (CDC) growth charts based on a more representative US population.^14^ However, the release of the new WHO child growth standards in 2006, which are based on selected multinational samples of children with minimal constraints to growth, provided a better standard for the growth of children below 5 years of age.^11^ Subsequently, the WHO recommended that this new standards be used for the surveillance of nutritional status in all countries and offered assistance for statistical analysis of data collected before the release of the new standards.

In this study, and in accordance with the WHO reccommendation, the WHO standards were used as a reference to calculate the prevalence of nutritional indicators in Saudi children. In addition, comparison with the prevalence data from other countries was made only with those using the same reference (WHO child growth standards) in a similar age group of children (birth to 5 years). The effect of the type of reference on the prevalence of nutritional indicators is well known and may be considerable. For example, the prevalence of underweight was higher in children under 2 years of age when using the WHO standards than when using the 1990 United Kingdom reference.^15^ Another report comparing WHO standards with the NCHS/WHO reference using data from the Dominican Republic, Bangladesh and pooled data from the United States and Europe found higher rates of underweight, stunting, and wasting when the WHO standards were used as reference.^16^ In addition, comparison between the WHO standards and the CDC reference revealed heavier and shorter children in the CDC sample.^17^

This report establishes the prevalence of underweight, wasting, and stunting in a representative national sample of Saudi children under 5 years of age and should serve as baseline for the surveillance of nutritional status in this population. The finding of a lower prevalence of all nutritional indicators in girls is consistent with results of all studies summarized inTable 4. This universal finding is most probably related to the different pattern of growth between boys and girls in this age group, charcterized by lower growth chart curves (–2 and –3 SD) for girls for all growth indices resulting in a smaller proportion of girls below the cut offs (–2 SDS or –3 SDS), leading to a lower prevalence for all indicators.^18^

The countries selected for the comparison inTable 4 are representative of economically developed, less developed (also called transitional), and least developed countries, so as to demonstrate that a huge disparity still persist in the 21st Century with about 40% prevalence of moderate underweight in Bangladesh^19^ and Yemen^20^ to 2.2% in Brazil^21^ and 1.3% in the USA.^17^ This large gap closely reflects economic development and resources. The prevalence of moderate underweight in Saudi children of 6.9% is closer to the 5.4% reported from Egypt^22^ indicating an intermediate position. The 9.8% prevalence of wasting in Saudi children, however, is closer to 11.2% rates reported from Nigeria^23^ and very far from rates reported from Indonesia.^24^ The 10.9% prevalence of moderate stunting is slightly lower than the 12.9% reported from Oman, while the prevalence of severe stunting is similar.^25^ The sources for the comparisons between countries were either the original data or a re-analysis according to WHO standards as presented in the WHO website Global Data.

In conclusion, the prevalence of underweight, wasting, and stunting in Saudi children is intermediate between socioeconomically developed and developing countries. However, these prevalence rates are still higher than “transitional” countries such as Brazil, indicating that more efforts are needed to improve child health in general and nutritional status in particular. Accordingly, it is recommended that regular surveillance in the form of nutritional surveys be conducted at the national level to monitor the nutritional health of Saudi children.
