# errata

**Published:** 2010

El Mouzan et al. Prevalence of malnutrition in Saudi children: a community-based study. Ann Saudi Med 2010;30(5):381-385. PMID: 20697172. Some data in some of the tables (and the corresponding data in the text) are corrected as indicated. Changes are highlighted.

**Table 1:** Prevalence of underweight by age and gender.

Age (years)	Total number (%) <-2 SD			Total number (%) <-3 SD		
	Boys	Girls	Combined	Boys	Girls	Combined
<1	4343 (5.1)	4141 (2.3)	8484 (3.7)	4343 (1.1)	4141 (0.5)	8484 (0.8)
1 to < 2	1283 (5.5)	1228 (2.9)	2511(4.2)	1283 (0.8)	1228 (0.4)	2511 (0.6)
2 to < 3	703 (7.8)	727 (7.2)	1430 (7.5)	703 (1)	727 (1.5)	1430 (1.3)
3 to < 4	716 (7.8)	787 (8.8)	1503 (8.3)	716 (2)	787 (1.5)	1503 (1.8)
4 to < 5	845 (10.3)	828 (11.1)	1677 (10.7)	845 (2.1)	828 (1.6)	1673 (1.9)
**Overall**	**7890**(6.1)^a^	**7711**(4.5)^a^	**15601**(5.3)	**7890**(1.2)^b^	**7711**(0.8)^b^	**15 601**(1.0)

a *P*<.0001, Significant difference in prevalence of moderate underweight between boys and girls (*P*<.0001).

b *P*< =.0154 Significant difference in prevalence of severe underweight between boys and girls (*P*=.0154).

**Table 2:** Prevalence of wasting by age and gender.

Age (years)	Total number (%) <-2 SD			Total number (%) <-3 SD		
	Boys	Girls	Combined	Boys	Girls	Combined
<1	4290 (12.3)	4109 (10.8)	8399 (11.6)	4290 (5.6)	4109 (4.4)	8399 (5)
1 to < 2	1282 (6.9)	1227 (4.5)	2509 (5.7)	1282 (1.9)	1227 (1.6)	2509 (1.8)
2 to < 3	698 (12.3)	727 (8.4)	1425 (10.4)	698 (3.2)	727 (2.8)	1425 (3)
3 to < 4	714 (10.5)	785 (9)	1499 (9.8)	714 (2.5)	785 (1.9)	1499 (2.2)
4 to < 5	893 (12.6)	826 (10.8)	1719 (11.7)	893 (3.5)	826 (1.9)	1719 (2.7)
**Overall**	**7823 (12.7)**^a^	**7674(10.8)**^a^	**15 497 (11.8)**	**7823 (5.1)**^a^	**7674 (3.9)**^a^	**15 497 (4.5)**

a *P*<.0001

**Table 3:** Prevalence of stunting by age and gender.

Age (years)	Total number (%) <-2 SD			Total number (%) <-3 SD		
	Boys	Girls	Combined	Boys	Girls	Combined
<1	4336 (10.5)	4123 (5.2)	8459 (7.9)	4336 (3.3)	4123 (1.5)	8459 (2.4)
1 to < 2	1278 (12.5)	1225 (11.1)	2503 (11.8)	1278 (3.8)	1225 (3.3)	2503 (3.6)
2 to < 3	703 (14.1)	726 (10.1)	1429 (12.1)	703 (3.4)	726 (3.4)	1429 (3.4)
3 to < 4	716 (11.7)	787 (12.8)	1503 (12.3)	716 (3.4)	787 (2.5)	1503 (3.0)
4 to < 5	845 (10.1)	829 (10.3)	1674 (10.2)	845 (1.8)	829 (1.9)	1674 (1.9)
**Overall**	**7878 (10.8)**^a^	**7690** (7.8)^a^	**15568** (9.3)	**7878** (3.1)^a^	**7690** (2.1)^a^	**15 568** (2.6)

a *P*<.0001

**Table 4:** Comparative national prevalence of nutritional indicators in selected countries.

Country	Study years	Sample size	Underweight (%)		Wasting (%)		Stunting (%)	
			←2 SD	←3 SD	←2 SD	←3 SD	←2 SD	←3 SD
Bangladesh^19^	2006	24 302	39.8	10.3	12.4	1.6	47	15.5
Yemen^20^	2003	12 364	43.1	18.5	15.2	6.3	57.7	35.8
Brazil^21^	2006-07	4415	2.2	0.3	4.0	1.6	7.1	1.5
USA^17^	1999-04	3920	1.3	0.2	0.6	0.1	3.9	0.5
Egypt^22^	2005	12 830	5.4	1.7	5.3	2.5	23.8	10.3
Nigeria^23^	2003	5407	27.9	11.9	11.2	4.8	43	23.7
Indonesia^24^	2007	77 808	19.6	5.1	14.8	6.8	40.1	21
Oman^25^	1999	14 076	11.3	1.6	7.3	1.1	12.9	2.4
Present study	2004-05	15 601	5.3	1.0	11.8	4.5	9.3	2.6

